# Clinical effect of non-convulsive electroconvulsive therapy combined with olanzapine in patients with schizophrenia

**DOI:** 10.3389/fneur.2025.1641908

**Published:** 2025-11-12

**Authors:** Kecai Tao, Qian Xiong, Jinghua Wu

**Affiliations:** 1 Mental Health Center, West China Hospital, Sichuan University, Chengdu, China; 2 West China School of Nursing, Sichuan University, Chengdu, China; 3 Department of Head and Neck Surgery, West China Tianfu Hospital of Sichuan University, Sichuan, Chengdu, China; 4 Department of Psychiatry, Dongguan No. 7 People’s Hospital, Dongguan, Guangdong, China

**Keywords:** schizophrenia, non-convulsive electroconvulsive therapy, olanzapine, cognitive function, BDNF

## Abstract

**Objective:**

To explore the clinical effect of non-convulsive electroconvulsive therapy combined with olanzapine in schizophrenia patients.

**Methods:**

From September 2021 to August 2023, a total of 112 patients with schizophrenia treated in our hospital were selected as the study participants. These patients were randomly divided into a control group and a research group, with 56 cases in each group. Patients in the control group were administered olanzapine tablets. Based on olanzapine tablets, patients in the research group received non-convulsive electroconvulsive therapy. The psychotic symptoms, severity of schizophrenia symptoms, memory ability, cognitive function, serum BDNF, S100B, and GFAP levels, and incidence of adverse reactions between the two groups were compared.

**Results:**

Compared to the control group, the research group had lower BPRS score, lower PANSS score, higher WMS-IV score, higher BACS score, higher serum BDNF level, lower serum S100B and GFAP levels (*p* < 0.05). However, there was no significant difference in the incidence of adverse reactions between the two groups (*p* > 0.05).

**Conclusion:**

Non-convulsive electroconvulsive therapy combined with olanzapine has a significant effect in the treatment of schizophrenia. It can effectively improve mental symptoms and cognitive function of patients, regulate the levels of serum BDNF and S100B, and has high safety.

## Introduction

Schizophrenia is a severe mental disorder, and its onset is related to multiple factors (1). Currently, the exact cause and mechanism of the disease are not fully understood, and it may be related to various factors such as the patient’s genetics, personality, upbringing environment, and living conditions (2). Most patients develop the disease during their youth, and the main clinical manifestations are the incoordination between mental activities and the environment, including disorders in sensation, thinking, emotion, and behavior, and some patients may experience cognitive function impairment during the disease progression (3). Statistical data show that the prevalence of schizophrenia worldwide is approximately 3%, and it is increasing year by year, imposing a heavy burden on families and society (4).

In clinical treatment, antipsychotic drugs are the main medication. The emergence of a large number of new drugs has improved the therapeutic effect, among which olanzapine can better relieve mental symptoms, and the has fewer adverse reactions when used for a long time, but its overall effect is still limited when used alone, and the quality of life of patients cannot be improved (5). Non-convulsive electroconvulsive therapy is a new treatment method, mainly through the combination with anesthetics and muscle relaxants, using specific pulse currents to stimulate the brain, causing the patient to lose consciousness for a certain period of time, regulating the release of neurotransmitters, and reducing the abnormal discharge threshold of the brain (6). Relevant studies abroad have confirmed that non-convulsive electroconvulsive therapy has high safety, simple operation, and significant effect, and is very suitable for the treatment of schizophrenia, depression and mania (7, 8).

In our study, we explored the clinical effect of non-convulsive electroconvulsive therapy combined with olanzapine in patients with schizophrenia.

## Materials and methods

### Study design

From September 2021 to August 2023, a total of 112 patients with schizophrenia treated in our hospital were selected as the study participants. These patients were randomly divided into a control group and a research group, with 56 cases in each group. The family members of all the patients fully understood the relevant content of this study and signed the corresponding consent forms. This study was approved by the ethics committee of Mental Health Center of Sichuan University Hospital. The clinical registration number was ChiCTR2100053045.

Inclusion criteria: (1) All patients in this study met the relevant diagnostic criteria in the “Chinese Classification and Diagnosis Criteria for Mental Disorders”; (2) The included patients had not taken antipsychotic drugs or drugs that interfered with thyroid hormone secretion in the past 2 months; Exclusion criteria: (1) Patients had severe liver and kidney dysfunction; (2) Allergic to the drugs used in this study; (3) Pregnant and lactating women; (4) Alcoholics, those with strong aggression, and those with organic brain lesions; (5) The patient withdrew from the treatment halfway.

### Randomization and blinding

**Figure 1:**
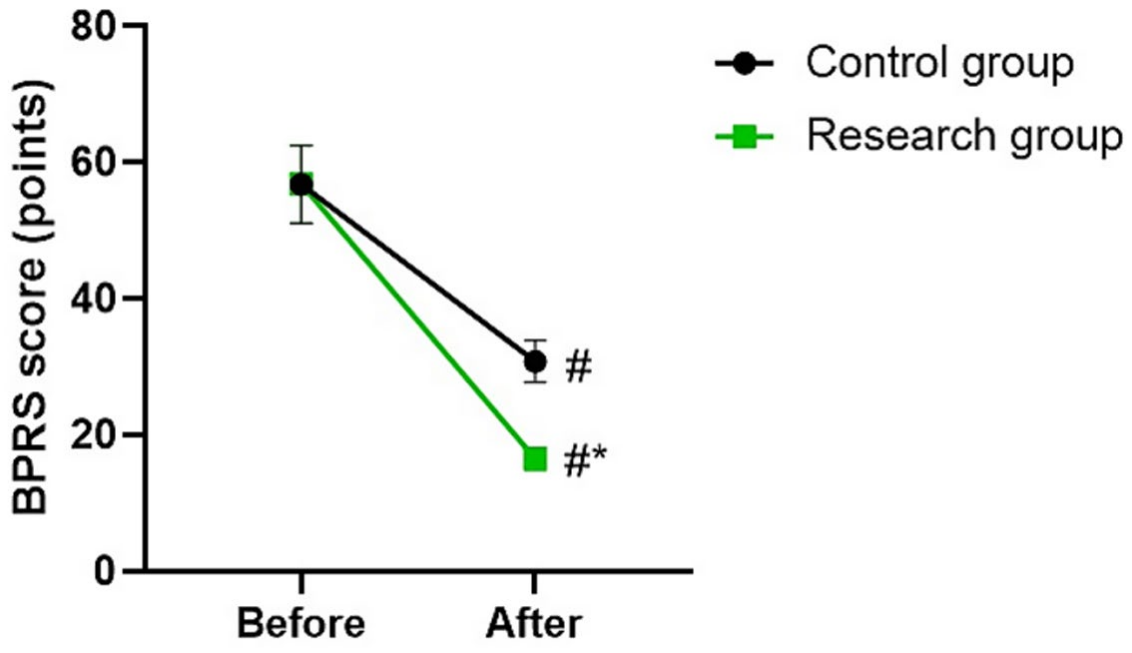
CONSORT flowchart.

A group randomization design was adopted for random grouping. The random allocation sequence was generated by a computer. The allocation confidentiality measures were achieved through sequential numbering, sealing, and opaque envelopes. After being deemed to meet the inclusion criteria, patients were randomly assigned to the control group or the research group in a 1:1 ratio (Figure 1). This study was single-blind, and the participants were unaware of the allocation.

### Methods

Patients in the control group were administered olanzapine tablets (manufacturer: Jiangsu Hausen Pharmaceutical Group Co., LTD.). The initial dose was 5 mg each time, once a day. After 7 days, the dosage was increased to 10 to 15 mg, once a day, and this dosage was maintained for 8 weeks.

Based on olanzapine tablets, patients in the research group received non-convulsive electroconvulsive therapy. The instrument used was the American Thymatron System IV electroconvulsive therapy instrument.

Before the treatment, the patient needed to stop drinking water and fast for 8 h. After the treatment began, the patient was injected with atropine (manufacturer: Chengdu First Pharmaceutical Co., LTD.) at a dose of 0.5 mg and propofol (manufacturer: Sichuan Guoruei Pharmaceutical Co., LTD.) at a dose of 1.0 mg/kg in sequence. When the ciliary reflex completely disappeared, the patient was intravenously injected with succinylcholine chloride (manufacturer: Shanghai Xudong Haipu Pharmaceutical Co., LTD.) at a dose of 1.0 mg/kg.

After the patient’s muscles fully relaxed, the non-convulsive electroconvulsive therapy was carried out. The pulse width was 0.5 milliseconds, the treatment frequency was set at 50 hertz, and each electrical stimulation lasted for 2 s. Before the treatment began, a threshold measurement was conducted first. This was done by gradually increasing the current intensity, starting from a lower value, and increasing it by 10–20 milliampere each time until typical epileptic-like discharges were observed in the patient. The current intensity at this point was the patient’s seizure threshold. In the subsequent treatment, the stimulation intensity was adjusted appropriately based on the patient’s individual response and treatment outcome. Generally, the stimulation intensity was set at 1.5 times the seizure threshold for the first treatment to ensure the treatment effect. As the treatment progresses, if the patient showed a good response and no obvious adverse reactions, the stimulation intensity was gradually adjusted to 1.2–1.3 times the seizure threshold; if the treatment effect was poor or the patient develops tolerance, the stimulation intensity was appropriately increased under the condition of ensuring safety, but not exceeding 2 times the seizure threshold.

Initially, the non-convulsive electroconvulsive therapy was administered three times a week. Starting from the third week, it was conducted once a week for a total of 8 weeks. Therefore, a total of 12 non-convulsive electroconvulsive therapy sessions were carried out.

### Observation indicators

1. The psychotic symptoms of patients were evaluated using the Brief Psychiatric Rating Scale (BPRS) (9). This scale included five aspects: anxiety and depression, lack of energy, thinking disorder, activation, and hostile suspicion. The higher the score, the more severe the symptoms.
2. The severity of schizophrenia symptoms was evaluated using the Positive and Negative Syndrome Scale (PANSS) (10). The scale consisted of 7 positive symptom items, 7 negative symptom items, and 14 general mental symptom items. The total score was positively correlated with the severity of schizophrenia symptoms.
3. The memory ability was assessed using the Wechsler Memory Scale-Fourth Edition (WMS-IV) (11). This scale included 10 test items, including experience, orientation, number sequence relationship, recognition, picture recall, visual regeneration, associative learning, tactile memory, logical memory and recitation number. The highest score was 121 points. The higher the score, the stronger the memory ability.
4. The cognitive function was assessed using the brief assessment of cognition in schizophrenia (BACS) (12). This scale included executive function, alertness, memory, verbal fluency, motor speed, working memory, and verbal memory. The higher the score, the stronger the cognitive ability.
5. In the morning, when the patient was in a fasting state, a sterile syringe was used to collect a 5 mL blood sample from the patient’s elbow vein. It is necessary to clarify the difference between serum and plasma: Serum is the pale-yellow transparent liquid separated from the blood after it coagulates, by removing fibrinogen and certain coagulation factors from the plasma; while plasma is the pale-yellow liquid separated after adding anticoagulants to the blood, containing fibrinogen and other coagulation factors. In this study, the collected blood samples were not treated with anticoagulants. They were placed at room temperature and left to stand for 30 min. After the blood naturally coagulated, the samples were centrifuged at a speed of 3,000 revolutions per minute for 15 min to separate the blood cells from the serum completely. Then, the clear yellow upper liquid, which is the serum, was carefully aspirated and transferred to a clean centrifuge tube. It was stored in a − 80 °C refrigerator for testing. The serum levels of brain-derived neurotrophic factor (BDNF), S100 calcium-binding protein beta subunit (S100B) and glial fibrillary acidic protein (GFAP) were detected by enzyme-linked immunosorbent assay (ELISA). The ELISA kit for BDNF was produced by Chuzhou Shinuoda Biological Technology Co., Ltd., and the kit number was SND-H179. The ELISA kit for S100B was produced by Shanghai Jingkang Biotechnology Engineering Co., Ltd., and the kit number was JK-(EA)-10210. The ELISA kit for GFAP was produced by Shanghai Yaji Biotechnology Co., Ltd., and the kit number was YS01569B.
6. During the treatment period for both groups of patients, adverse reactions including abnormal heart rate, nausea and vomiting, dizziness and headache were recorded, and the overall incidence rates were compared.

### Statistical analysis

SPSS 24.0 statistical software was adopted for data analysis. Before conducting the formal statistical analysis, the Shapiro–Wilk test was used to determine whether the data followed a normal distribution. Measurement data were expressed as mean ± standard deviation (x ± s). Between-group comparisons were performed using independent t tests for continuous variables with normal distributions and Mann–Whitney U tests for data not normally distributed. A repeated-measures analyses of variance was performed with a within-group factor of time (before vs. after treatment). Count data were expressed as (n, %), and *χ*^2^ test was used for comparison. Effect sizes were presented as 95% confidence intervals (CI). *p* < 0.05 meant the difference was statistical significance.

## Results

### General data between the two groups

In the control group, there were 24 males and 32 females, aged from 22 to 50 years, with an average age of (34.35 ± 3.42) years. The course of disease ranged from 6 months to 3 years, with an average of (1.72 ± 0.53) years. In the research group, there were 25 males and 31 females, aged from 23 to 52 years, with an average age of (34.42 ± 3.46) years. The course of disease ranged from 7 months to 3 years, with an average of (1.75 ± 0.56) years. There was no statistical significance in general data between the two groups (*p* > 0.05; Table 1), indicating comparability.

**Table 1:** General data of patients between the two groups.

Items	Control group (*n* = 56)	Research group (*n* = 56)	*χ*^2^/*t*	*p*	95% CI
Gender			0.036	0.848	0.654–1.395
Male	24 (42.86)	25 (44.64)			
Female	32 (57.14)	31 (55.36)			
Age (years)	34.35 ± 3.42	34.42 ± 3.46	0.107	0.914	−1.218-1.358
Course of disease (years)	1.72 ± 0.53	1.75 ± 0.56	0.291	0.771	−0.174-0.234

### BPRS score between the two groups

**Figure 2:**
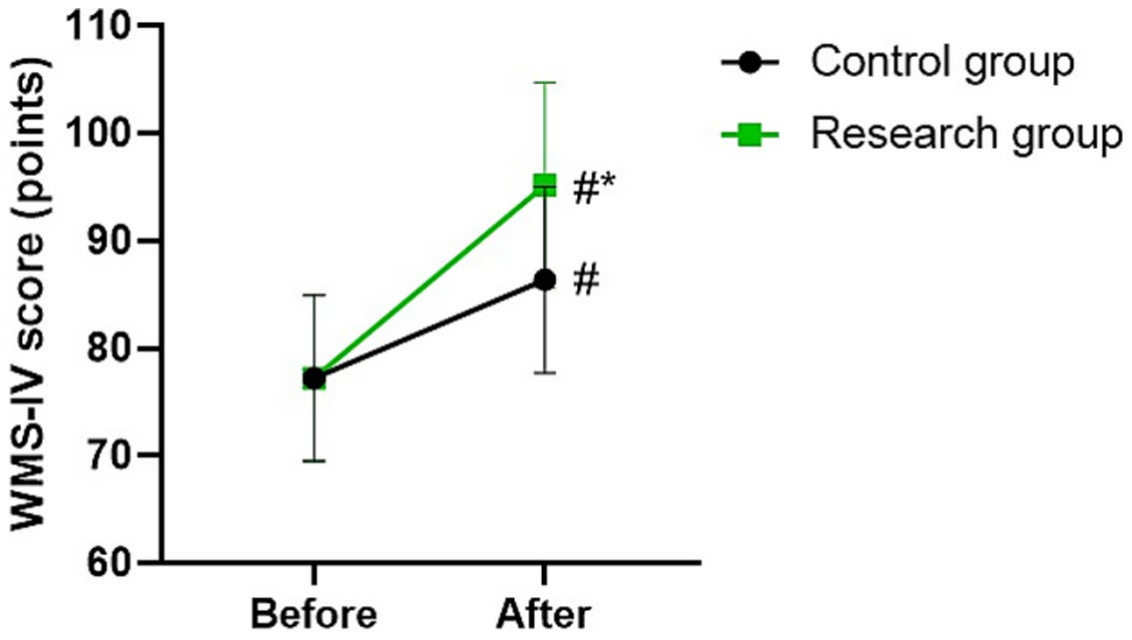
BPRS score between the two groups. ^#^*p* < 0.05, compared with before therapy. ^*^p < 0.05, compared with CG.

Before therapy, there was no difference in BPRS score between the two groups (*p* > 0.05). The BPRS scores of the two groups were lower after therapy than those before therapy (*p* < 0.05). Compared with the control group, the research group had lower BPRS score after therapy (*p* < 0.05; Figure 2).

### PANSS scores in 2 groups

**Figure 3:**
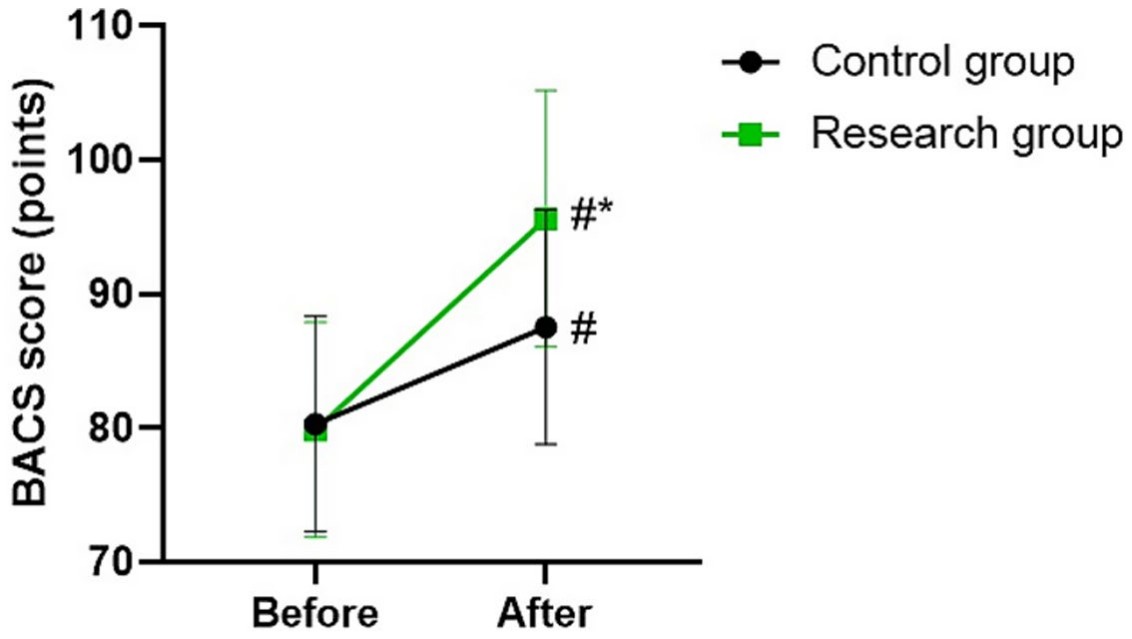
PANSS scores between the two groups. ^#^*p* < 0.05, compared with before therapy. ^*^p < 0.05, compared with CG.

Before therapy, there were no differences in PANSS scores between the two groups (*p* > 0.05). The PANSS scores of the two groups were lower after therapy than those before therapy (*p* < 0.05). Compared with the control group, the research group had lower PANSS scores after therapy (*p* < 0.05; Figure 3).

### WMS-IV score in 2 groups

**Figure 4:**
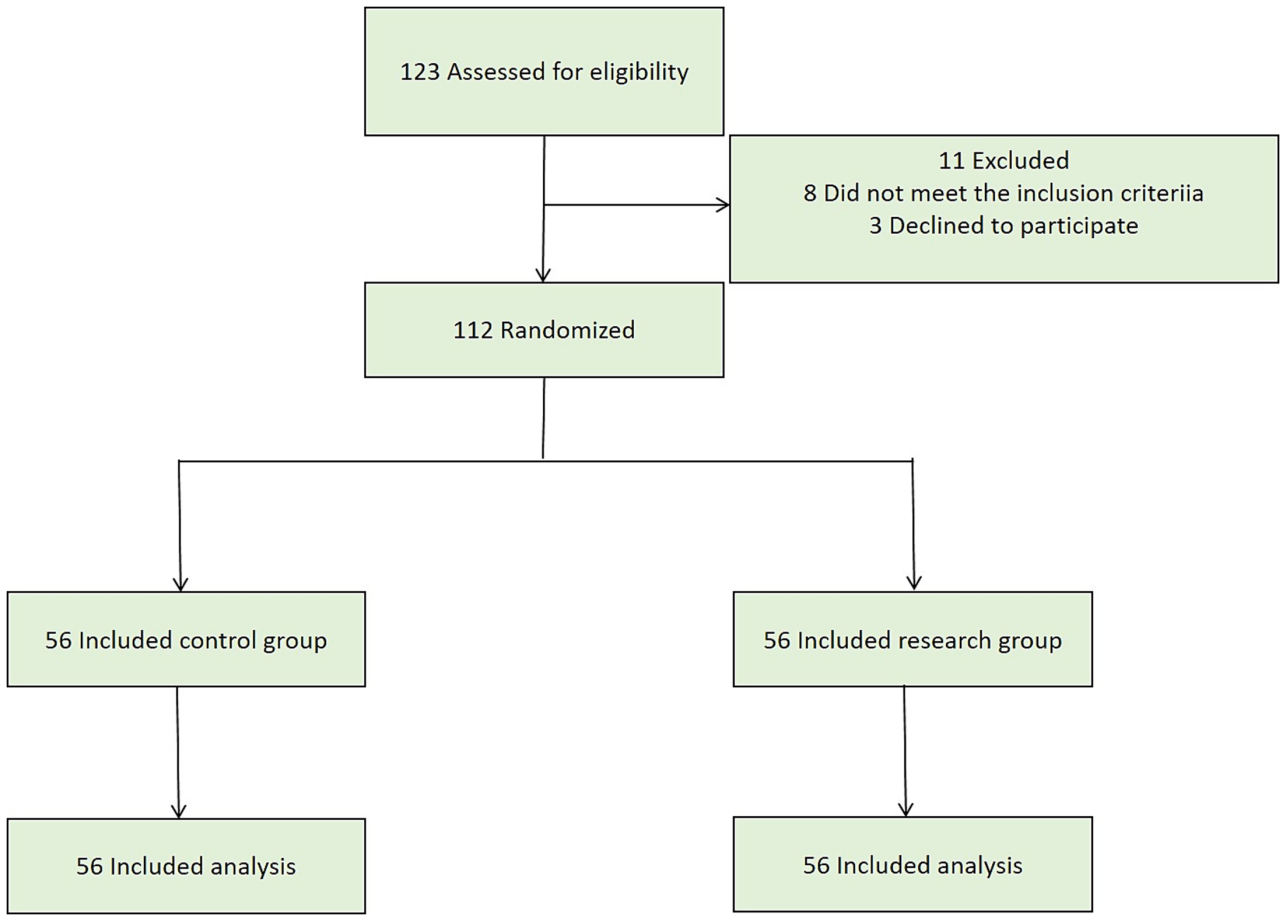
WMS-IV score between the two groups. ^#^*p* < 0.05, compared with before therapy. ^*^p < 0.05, compared with CG.

Before therapy, there was no difference in WMS-IV score between the two groups (*p* > 0.05). The WMS-IV scores of the two groups were higher after therapy than those before therapy (*p* < 0.05). Compared with the control group, the research group had higher WMS-IV score after therapy (*p* < 0.05; Figure 4).

### BACS score in 2 groups

**Figure 5:**
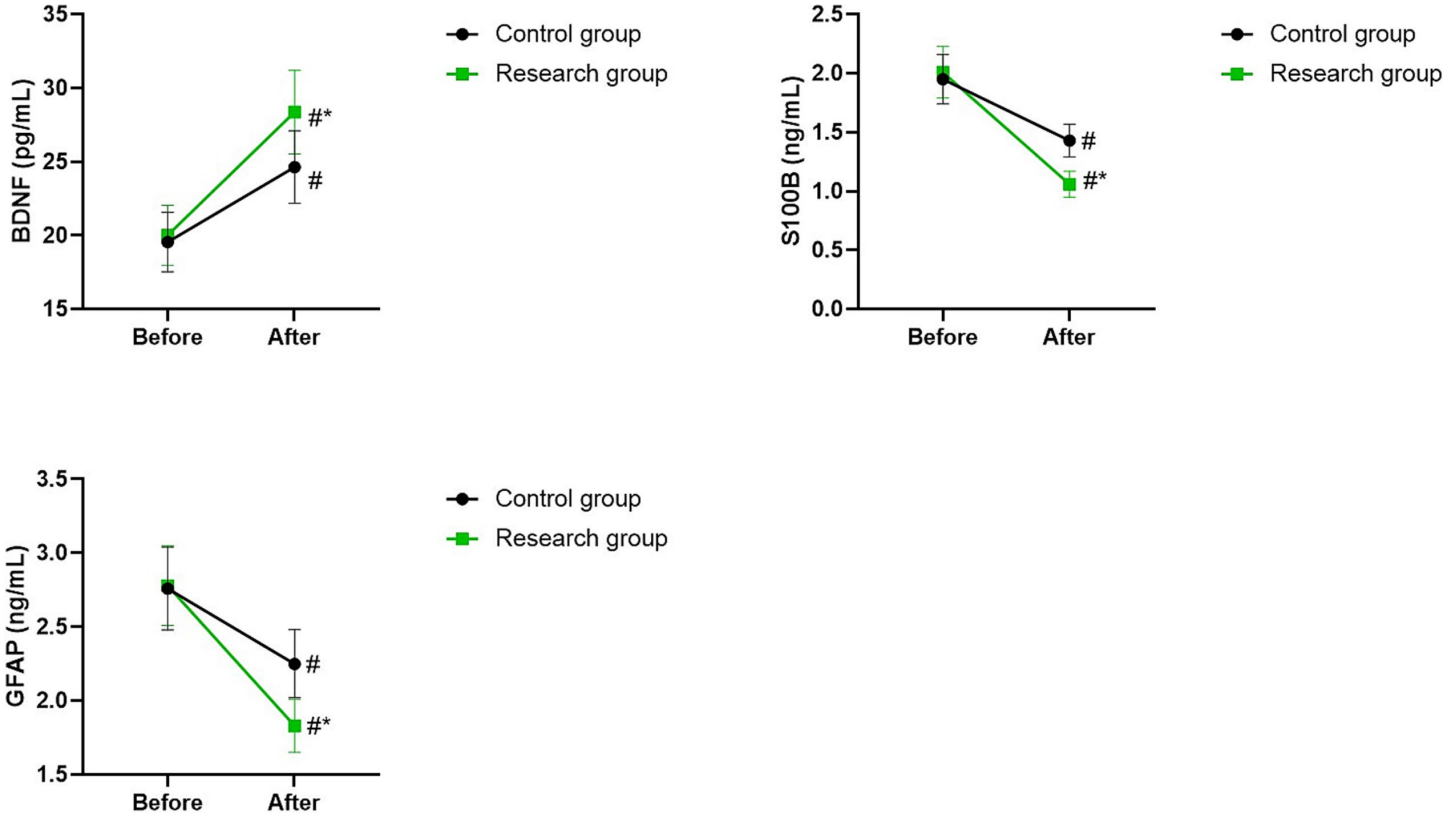
BACS score between the two groups. ^#^*p* < 0.05, compared with before therapy. ^*^p < 0.05, compared with CG.

Before therapy, there was no difference in BACS score between the two groups (*p* > 0.05). The BACS scores of the two groups were higher after therapy than those before therapy (*p* < 0.05). Compared with the control group, the research group had higher BACS score after therapy (*p* < 0.05; Figure 5).

### Serum BDNF, S100B, and GFAP levels in 2 groups

**Figure 6:**
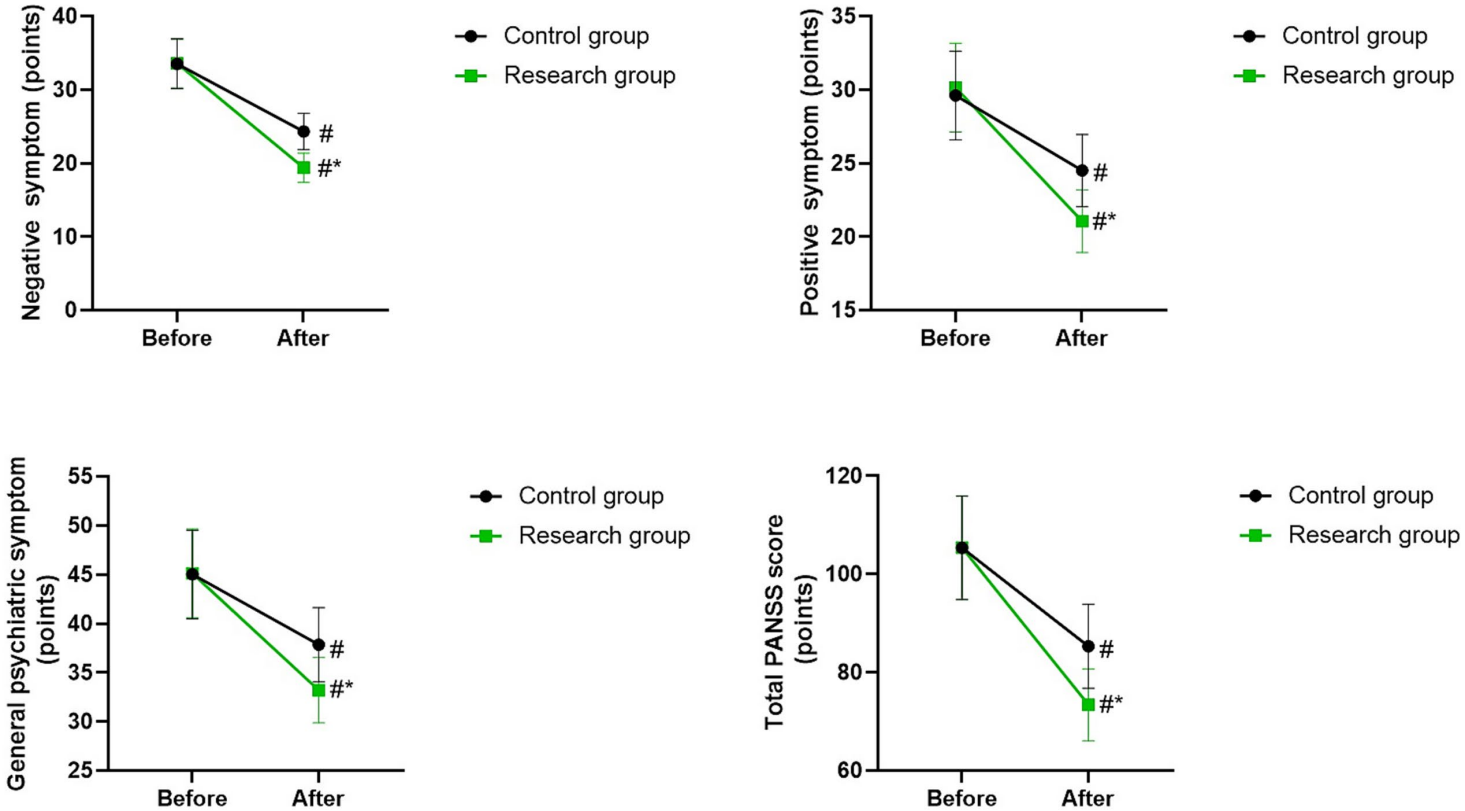
Serum BDNF, S100B, and GFAP levels between the two groups. ^#^*p* < 0.05, compared with before therapy. ^*^p < 0.05, compared with CG.

Before therapy, there were no differences in serum levels of BDNF, S100B, and GFAP between the two groups (*p* > 0.05). The serum levels of BDNF of the two groups were higher after therapy than those before therapy, while the serum levels of S100B and GFAP of the two groups were lower after therapy than those before therapy (*p* < 0.05). Compared with the control group, the research group had higher serum level of BDNF as well as lower serum levels of S100B and GFAP after therapy (*p* < 0.05; Figure 6).

### Incidence of adverse reactions in 2 groups

There was no significant difference in the incidence of adverse reactions between the two groups (*p* > 0.05; Table 2).

**Table 2:** Incidence of adverse reactions between the two groups.

Groups	Cases	Abnormal heart rate	Nausea and vomiting	Dizziness and headache	Total incidence rate
Control group	56	2 (3.57)	2 (3.57)	2 (3.57)	6 (10.71)
Research group	56	2 (3.57)	3 (5.36)	2 (3.57)	7 (12.50)
*χ* ^2^					0.087
*p*					0.768
95% CI					0.449–1.493

## Discussion

Schizophrenia is one of the common severe diseases in psychiatric outpatient clinics. Its causes are complex and the course of disease is long. The emotional, thinking, cognitive and behavioral functions of patients have all been severely impaired, and there is a possibility of deterioration and chronicity (13). If effective treatment measures are not taken in time, it will not only threaten the health and safety of the patients themselves, but also easily affect social order (14).

Currently, the clinical treatment of schizophrenia mainly relies on antipsychotic drugs (15). Olanzapine is one of the commonly used drugs in clinical practice. It can exert pharmacological effects on a variety of receptor systems, regulate the binding effect of dopaminergic D1 and D2 receptors, and antagonize 5-HT. (16) Compared with other nerveblocking drugs, olanzapine has little impact on receptor binding functions and can reduce the probability of adverse reactions (17). However, according to relevant clinical studies, olanzapine can improve the negative and positive symptoms of patients with refractory schizophrenia, but its regulatory effect on the cognitive function of patients is not very significant (18).

Non-convulsive electroconvulsive therapy belongs to the optimized electroconvulsive therapy intervention mode (19). Compared with previous electroconvulsive therapy, before electroconvulsive therapy intervention, appropriate anesthetic and muscle relaxant will be injected in advance to make patients to fall asleep quickly, keep the muscles relaxed, eliminate the patient’s negative psychological emotions such as tension and anxiety, and reduce the occurrence of muscle convulsions (7). Then, a moderate current is applied to stimulate of the patient’s brain, causing the patient’s consciousness to gradually lose, thereby achieving the purpose of non-epileptic treatment and improving the symptoms of patients with schizophrenia (20).

In our study, the results showed that after therapy, the BPRS score and PANSS score of the research group were lower than those of the control group, while the WMS-RS score and BACS score of the research group were higher than those of the control group. These results suggested that non-convulsive electroconvulsive therapy combined with olanzapine could better improve the psychotic symptoms, reduce the severity of schizophrenia symptoms, promote the memory ability and cognitive function of schizophrenia patients. Generally speaking, traditional electroconvulsive therapy does cause some cognitive side effects. Among them, memory decline is a relatively common issue (21). This is mainly because during the treatment process of traditional electroconvulsive therapy, the larger current stimulation may have a relatively strong impact on the brain nerve cells, interfering with the normal transmission of neurotransmitters and the normal connection of neural networks, thereby affecting the functions of brain regions such as the hippocampus, which are closely related to memory formation and storage, leading to memory disorders such as anterograde amnesia and retrograde amnesia in patients. However, the non-convulsive electroconvulsive therapy employed in this study differs significantly from the traditional electroconvulsive therapy. Non-convulsive electroconvulsive therapy is a modified form of electroconvulsive therapy that precisely controls parameters such as reducing current intensity, adjusting pulse frequency and width. This allows the electrical stimulation to reach the effective dose required to treat psychiatric symptoms while minimizing excessive stimulation of brain nerve cells. This gentle stimulation method may avoid the excessive damage to key brain regions such as the hippocampus caused by traditional electroconvulsive therapy, thereby reducing the negative impact on memory function (22). In addition, the possible mechanisms by which non-episodic electroconvulsive therapy improves cognitive function include: (1) Non-convulsive electroconvulsive therapy may reduce the tightness of the blood–brain barrier by altering the expression and distribution of tight junction proteins on the barrier. This enables the drug molecules of olanzapine to penetrate the cerebrospinal fluid more effectively. When the concentration of olanzapine in the brain reaches the therapeutic level, it can exert its antipsychotic effect more fully, regulate the balance of neurotransmitters, improve the functional state of neurons, and thereby indirectly promote the recovery of cognitive functions (23); (2) Non-convulsive electroconvulsive therapy may promote the survival, growth and differentiation of neurons by activating the expression of neurotrophic factors such as NGF and BDNF, increase the number and density of synapses, and improve the connectivity and function of neural networks; (3) Non-convulsive electroconvulsive therapy may have the effect of regulating the inflammatory response of the nervous system. By inhibiting the excessive activation of microglia cells, it reduces the release of inflammatory factors such as tumor necrosis factor-*α* (TNF-α) and interleukin-6 (IL-6), alleviates the damage caused by neural inflammation to neurons, protects the normal functions of neurons, and thereby promotes the improvement of cognitive function (24). Consistently, Yin et al. indicated that non-convulsive electroconvulsive therapy combined with escitalopram can reduce depressive symptoms, promote the quality of life, and improve the cognitive function of patients with treatment-resistant depression (25).

BDNF is an indispensable neurotrophic factor for neurodevelopment, widely present in the cerebral cortex, cerebellum, and hippocampus (26, 27). It not only regulates the cognitive function of the brain, but also participates in the learning and memory mechanism, and provides protection for neurons (28, 29). S100B is a neurotrophic factor synthesized by astrocytes, which can exert autocrine and paracrine effects in microglia and neurons (30, 31). High concentration of S100B can induce programmed death of nerve cells and lead to nerve damage (30). GFAP is a specific protein of astrocytes, whose main function is to maintain the normal morphology and function of astrocytes, but astrocytes have a bidirectional nature, and excessive activation can cause damage to neurons (32, 33). Previous studies have shown that the serum BDNF level of schizophrenia patients is lower than that of healthy individuals, while the levels of S100B and GFAP are higher than those of healthy individuals, and can be used as markers of nerve damage (34–36). In this study, the serum level of BDNF of the research group was significantly higher than that of the control group, while the serum levels of S100B and GFAP in the research group were significantly lower than those in the control group. These results suggested that BDNF, S100B and GFAP may be involved in the mechanism of non-convulsive electroconvulsive therapy in the treatment of schizophrenia. Most current studies have shown that the therapeutic mechanism of non-convulsive electroconvulsive therapy may be related to neuroendocrinology, with the PI3K-mediated signaling pathways playing a key role, and neurotrophic factors such as BDNF, S100B, and GFAP play a mediating role in these pathways and can protect neurons from damage (37). Additionally, our study showed that there was no significant difference in the incidence of adverse reactions between the two groups, indicating that olanzapine combined with non-convulsive electroconvulsive therapy had a higher safety in the treatment of schizophrenia.

Ahmed et al. performed a meta-analysis and indicated that in the treatment of treatment resistant schizophrenia, the combination of electroconvulsive therapy and clozapine yielded the best outcome (38). This finding provides us with a valuable perspective for comparing and deeply reflecting on the current research results. Firstly, it is necessary to clarify that there are significant differences in the treatment approaches between this study and the studies in the meta-analysis. This study focuses on the combined application of non-convulsive electroconvulsive therapy and olanzapine, while the meta-analysis emphasizes the combination of traditional electroconvulsive therapy and clozapine. Non-convulsive electroconvulsive therapy, as a modified form of electroconvulsive therapy, significantly reduces convulsions and loss of consciousness during the treatment process, thereby improving patient tolerance and safety. This aspect is fully demonstrated in the current study, where non-convulsive electroconvulsive therapy combined with olanzapine not only effectively improves patients’ psychological symptoms and cognitive functions, but also shows high safety. Secondly, from the perspective of drug selection, olanzapine and clozapine both belong to the second-generation antipsychotic drugs, but they differ in their pharmacological properties, efficacy, and side effects. Olanzapine, with its broad receptor action spectrum and good tolerance, is widely used in clinical practice. While clozapine is regarded as the “gold standard” due to its significant efficacy in treating refractory schizophrenia, it also comes with a higher risk of side effects, such as granulocytopenia. Therefore, this study chose olanzapine as the combined medication, aiming to explore a safer and more effective treatment plan, especially for patients who are not refractory or do not tolerate clozapine. Furthermore, regarding the therapeutic effect, the meta-analysis emphasized the outstanding performance of electroconvulsive therapy combined with clozapine in treating refractory schizophrenia. This is mainly attributed to the potent antagonistic effect of clozapine on the dopamine D4 receptor and the 5-HT2A receptor, as well as the rapid regulatory effect of electroconvulsive therapy on the brain neurotransmitter system. In this study, non-convulsive electroconvulsive therapy combined with olanzapine also demonstrated significant efficacy. Not only did it achieve positive results in the improvement of psychological symptoms and cognitive functions, but it also provided new insights into the biological mechanism of schizophrenia by regulating serum BDNF and S100B levels. This indicates that even without using clozapine, by optimizing electroconvulsive therapy techniques (such as non-convulsive electroconvulsive therapy) and combining appropriate antipsychotic drugs, similar or even better therapeutic effects can be achieved, in some cases even superior to those achieved with clozapine. Therefore, although the meta-analysis provided effective evidence for the combined use of electroconvulsive therapy and clozapine in the treatment of treatment resistant schizophrenia, this study demonstrated significant efficacy and high safety in non-refractory schizophrenia patients through the combined application of non-convulsive electroconvulsive therapy and olanzapine, offering another valuable option for clinical treatment. Future research can further explore the effects of different electroconvulsive therapy techniques (including non-convulsive electroconvulsive therapy) combined with various second-generation antipsychotic drugs, with the aim of providing more personalized, safe, and effective treatment options for patients with schizophrenia.

In conclusion, non-convulsive electroconvulsive therapy combined with drug therapy has a significant effect in the therapy of schizophrenia, which can effectively improve psychiatric symptoms and cognitive function of patients, regulate serum BDNF and S100B levels, and is safe.

## Data Availability

The datasets presented in this study can be found in online repositories. The names of the repository/repositories and accession number(s) can be found in the article/supplementary material.
